# In-situ partial reduction of biochar by overlaying a syngas stream

**DOI:** 10.1016/j.btre.2025.e00892

**Published:** 2025-04-02

**Authors:** Valentin Chataigner, Dominique Tarlet, François Ricoul, Jérôme Bellettre

**Affiliations:** a Florentaise, Le Grand Pâtis, 44850 Saint Mars du Désert, France; b Nantes Université, Laboratoire de Thermique et Energie de Nantes LTeN UMR CNRS 6607, France; c S3d Ingénierie, Groupe Keran 4 Rue René Viviani, 44200 Nantes, France

**Keywords:** Partial reduction, Activation, Pine bark, High available surface biochar

## Abstract

This work focuses on the partial reduction of a hot biochar by a hot syngas coming out of the partial oxidation zone in the Ariane® pyrolysis prototype. The patented geometry of this pyrolyser, with separate pyrolysis and partial oxidation zones, enables easy management of biochar and syngas flows. The aim is to increase the specific surface area of the biochar to achieve more agronomic specifications, while maintaining a high carbon content in the biochar. The experimental implementation of this partial reduction is achieved by adding a partial reduction zone at the bottom of the reactor, enabling direct contact between biochar and syngas. This part now enables biochar and syngas streams to be mixed in a high-temperature zone ($>500^{\circ }C$). The addition of this part increased the specific surface area of the biochar by $250\;m^{2}/g$ and preserved the carbon content of the biochar regardless of the biomass flow rate. Around 20% of the carbon in the biochar is consumed by the reduction and transferred from the biochar to the syngas. The syngas is thus enriched in particular in $H_{2}$ and $CO_{2}$ and depleted in $CH_{4}$, which opens up a wider range of development opportunities for this gas (biomethane, biohydrogen, biokerosene, cogeneration, etc.). Thanks to this activation method, a better compromise has been achieved (compared with the previous version of the prototype) between quality (specific surface area and carbon content) and quantity of biochar produced (anhydrous yield).

## Highlights

• Testing on a patented continuous, vertical and coaxial pyrolyzer.
• In-situ partial reduction of a post-pyrolysis hot biochar under combined $CO_{2}$ and steam flows.
• Specific surface of biochar increases strongly.

## 1 Introduction

Current environmental and climate challenges are driving the biochar industry forward. Biochar converts the organic carbon in biomass into a stable form, fixed carbon, and can therefore respond to environmental challenges. To develop in a sustainable way, the biochar sector needs to consider the supply and origin of the biomass, the industrial process for thermochemically converting biomass into biochar and the ways in which the biochar can be used. Pyrolysis can be carried out with a multitude of materials such as agricultural waste [1], sunflower edible oil waste [2], pine needles [3] or bovine manure [4], and can produce high added-value products such as biodiesel [1], bioinsecticides [2], biochars to improve anaerobic digestion [5], etc. from its solid, liquid and gaseous co-products. The agronomic outlet stands out for the many interactions between soils and biochar. Biochar provides the agronomic sector with potential solutions to a range of problems, including water retention, soil compaction, and the remediation of polluted soils [6].

This agronomic valorisation of biochar requires it to be functionalised. Functionalisation means researching and developing one or more specific characteristics of biochar with a view to its specific use. Biochar functions are the non-exhaustive set of physico-chemical parameters that describe it. These functions may be its density, porosity, cation exchange capacity, pH, particle size distribution, specific surface area, fixed carbon content, etc. Depending on the biochar's intended application, certain parameters need to be developed and controlled. These different functions are developed to varying degrees, depending on the parameters applied to the pyrolysis or activation process (residence time, temperature, heating rate, type of oxidising agent, etc.). The present work objective is to understand the relationships between reduction parameters and biochar functions. These relationships also depend on the biomass (nature, structure, composition, etc.). In this work, the influence of reduction parameters on the specific surface area and fixed carbon rate functions is investigated on a pine bark biochar. A high specific surface area is sought to increase exchanges between the biochar and the soil for agronomic applications including water retaining, nutrients capture and pollutants adsorption.

The first phase of this work (see [7]), using a pyrolysis prototype with separate oxidation and pyrolysis zones, produced a biochar with a specific surface area of 200 $m^{2}/g$ (for a 20 kg/h pine bark flow at 15 % moisture content). This specific surface function was linked to the pyrolysis parameters (residence time, pyrolysis temperature and heating rate). The fixed carbon content was around 90 % on average, with no observed dependence on the biomass flow rate. Obtaining a specific surface area of 200 $m^{2}/g$ by pyrolysis alone is an encouraging result and confirms that pine bark with a lignin content of 44% and a carbon content of 55 % is suitable for developing this function. Moreover, the anhydrous biochar yield was relatively high at 35 to 40%.

In the literature, the increase in the specific surface area of biochar is defined by the term "activation". Many authors have dealt with this subject, with widely varying results depending on activation conditions [[8], [9], [10], [11], [12]]). The concept of partial reduction (activation) of biochar in-situ by hot syngas [8] is taken up in this work but is taken a step further. In this work, syngas is not simply brought into contact with hot biochar, but a flow of syngas passes through a bed of hot biochar. In this way, the composition of the syngas entering this zone is constant and makes it possible to have regular partial reduction conditions. The level of thermochemical partial reduction reactions is therefore constant. The in-situ partial reduction conditions were modulated according to variations in biomass input rates ranging from 11 to 24 *kg/h*. As the specific surface area depends on several parameters, including the type of biomass, the method developed by Langlois [8] was adapted to our prototype by activating a pine bark biochar under a flow of hot syngas to reach at least 300 $m^{2}/g$ of surface area without affecting the carbon content with physical activation. Physical activation can be carried out with other pure oxidizing molecules such as $CO_{2}$ or $H_{2}O$. The aim of this method is to use only the raw elements generated by pyrolysis, without adding any external products. This is why syngas was used to activate the biochar. There is another main method for activating biochar, chemical activation. This method uses chemical substances (acids, bases, metal salts, etc.) to consume some of the carbon, cause heating and release pores. This method is more effective than physical activation on the specific surface, but its environmental impact is significant in terms of the production of these chemical elements and their treatment after activation.

As tested in this work, physical activation reduces the energy yield of biochar to the benefit of syngas. This energy transfer is facilitated through the carbon consumed by the biochar, which ends up in the syngas. However, the industrial deployment of this physical activation method, as tested in this research, remains a real challenge.

The objective of this research is therefore to experiment with the physical activation of a hot biochar under a syngas stream using an existing prototype. Its impact on the properties of the biochar (specific surface area and carbon content) and on the anhydrous biochar yield will be measured by comparing the results of the two versions of the prototype.

## 2 Materials and methods

### 2.1 Design process for the new pyrolysis pilot unit

**Fig. 1:**
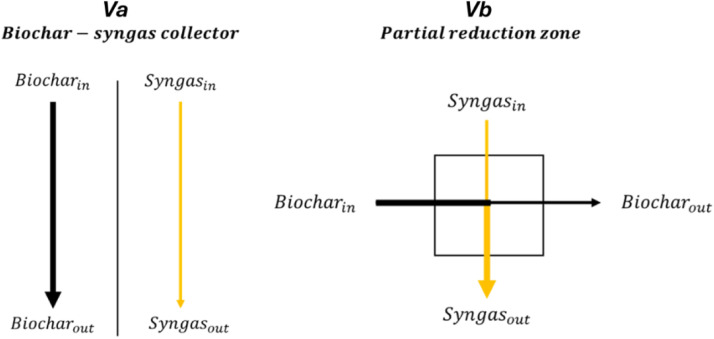
Biochar-syngas collector (Va: Version a (separated flows)) and partial reduction zone (Vb: Version b (mixed flows)).

To carry out pyrolysis and partial reduction of the biochar in the reactor without adding an activation stage outside the process, the biochar collector is replaced by a reduction zone. The aim is to create an activation zone by overlapping the syngas in the reduction zone. Inother words, this process creates mixed flows of biochar and syngas from the partial combustion of pyrolysis gases (Fig. 1). This modification must result in only partial reduction to contain the loss of biochar mass yield and not degrade its structure. The biochar collector initially installed on the pyrolyser must therefore be removed, replacing it with the reduction zone.

With this method, the quantity of gas produced should be greater than with conventional pyrolysis. The extra gas produced will pass directly through the syngas pipe without passing through the furnace [7], which is not harmful since the pyrolysis gases passing through the furnace are already surplus to the reactor's energy autonomy.

In addition to this main modification, a cyclone separator has been added to the syngas line to remove potential biochar particles from the gas stream and limit fouling of the heat exchanger, which cools the syngas and condenses the tars. Pressure probes are positioned at strategic points to monitor system fouling on different sections of the syngas line. Thermocouples are positioned in the reduction zone with many of these probes in identical positions to those they occupied in the biochar syngas collectors. The goal is to have a direct temperature comparison between the two versions of the pyrolyser.

### 2.2 Testing strategy

The definition of a test strategy is essential for this type of system. This strategy is based on the experience of the previous version of the pyrolyser [7].

The modification of the prototype by this new component has been the subject of a new phase of tests in order, firstly, to validate the new operation and, secondly, to determine the new range of bark flow rates that can be admitted into the reactor. The influence of this partial reduction zone will be evaluated, taking care to maintain pyrolysis conditions identical to the first test phase (without this reduction zone, [7]).

The range of throughputs tested will therefore remain in the same range as before, with an average throughput of bark (at 15 % moisture) varying from 10 to 30 $kg.h^{-1}$. Repeatability tests will also be carried out to determine whether the quality of the biochar remains unchanged under the same test conditions. Operating indicators (biochar yield temperatures, syngas composition, etc.) will be compared with the first experimental phase.

### 2.3 Influencing parameters for carbon – syngas reactions

There are many parameters influencing biochar reduction kinetics, some of them are similar to those for pyrolysis. Wang and Kinoshita [13] identify that the reduction temperature has an influence on the composition of the syngas and on the fraction of carbon remaining in the biochar, with a decrease in the intensity of the reaction when the carbon is almost completely consumed. Syngas composition and carbon conversion are also influenced by biochar residence time as observed by Chen et al. [14]. Finally, the size of the biochar particles affects the conversion rate of the biochar. Hernández et al. [15] found that the size of biochar particles should be between 2 and 4 mm for optimum ash/fixed carbon/volatile matter distribution. Other parameters are specific to biochar reduction, such as the composition of the oxidising gas. Shayan et al. [16] observe that steam increases more the volume fraction of $H_{2}$ than other oxidising gases (air and oxygen). Jayaraman et al. [17] compare the reduction with steam and $CO_{2}$ for different heating rates, which is also an influential parameter for reduction. They found that the kinetics of the steam reaction were faster than those of the $CO_{2}$ reaction. The concentration of oxidising agent also increases the rate of the reduction reaction, as noted by Huo et al. [18] with steam reduction. Finally, the air factor, studied by Puig-Arnavat et al. [19] and Ghassemi and Shahsavan-Markadeh [20] observe an optimum, since too much air factor dilutes the syngas with nitrogen and lowers its LHV even if it initially speeds up the reaction.

Finally, this experimental study will aim to determine the influence of partial reduction on the mass-energy balance and, more specifically, the anhydrous biochar yield and syngas composition. The quality of the biochar will also be analysed, in particular its specific surface area, carbon content and ash content. The modifications made to the pyrolyser are significant. As a result, new operating points must be obtained, in particular the air supply in relation to the bark flow rate (air factor). The range of biomass flow rates accepted by this new version has evolved somewhat, with flow rates ranging from 11 to 24kg/h, compared with 14 to 30kg/h for the previous version. Four common flow rates were obtained between the two versions (14, 17, 20 and 24kg/h). An analysis of the results for these four common flow rates is given in the following section.

## 3 Experimental tests

### 3.1 Experimental conditions

The thermokinetic conditions for pyrolysis on the new version of the prototype (temperatures, residence times, heating rates) are sufficiently similar to suggest that the pyrolysis mass-energy balances are similar between the two versions for 4 pine bark flow rates (14, 17, 20 and 24 kg/h). The reduction zone will therefore be the only vector between the already known characteristics of the biochar from the previous version and future analyses of the biochar from the new version of the pyrolyser.

The recirculation of syngas in the biochar bed increases the temperature by an average of $200^{\circ }C$. This increase is due to the syngas passing through the biochar bed, which transfers thermal energy to the solid carbon. Gas recirculation therefore enables the biochar to remain at the temperature at which it leaves the pyrolysis zone and to compensate for the endothermic effect of the partial reduction reaction by $CO_{2}$ and $H_{2}O$ according to an oxidising agent-solid carbon exchange. The activation temperature is measured at around 600 °C in the biochar reduction zone, allowing partial consumption of the biochar carbon and enrichment of the syngas.

The residence time of syngas in the reduction zone is of the order of a second, while the residence time of biochar in the reduction zone varies from 80 to 60 min for biomass flow rates ranging from 14 to 24kg/h.

### 3.2 Biochar anhydrous yield and syngas composition

**Fig. 2:**
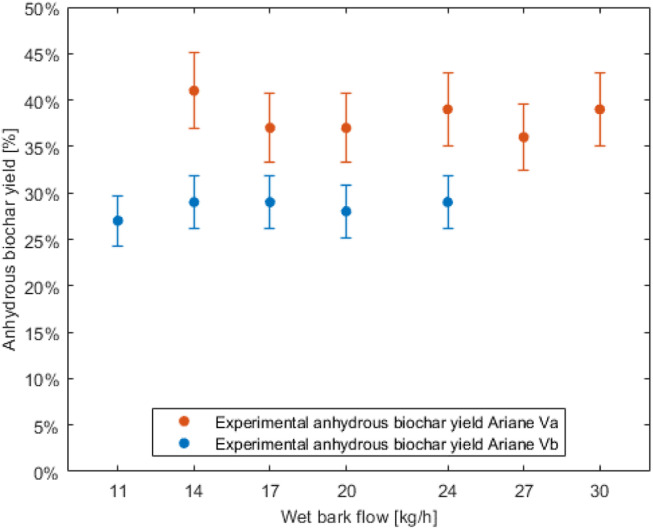
Anhydrous biochar yield comparison depending on wet bark flow rate.

The anhydrous yield of biochar shows a large difference between the two versions of the prototype, as shown in Fig. 2. As the thermokinetic conditions for pyrolysis are similar, this difference in anhydrous biochar yield is due essentially to the recirculation of syngas through the biochar bed in the partial reduction zone. The anhydrous yield of biochar without recirculation of syngas in the biochar bed is on average 38 % while with recirculation of syngas in the biochar bed, the anhydrous biochar yield averages 28 %.

In both versions of the pyrolyser, there is no discernible trend in biochar yield as a function of biomass throughput. This yield stability can be explained by reduction conditions (temperatures, concentration of gasifying agents, thermochemical exchanges, etc.) that are sufficiently independent of the biomass flow rate for no trend to be observed.

**Fig. 3:**
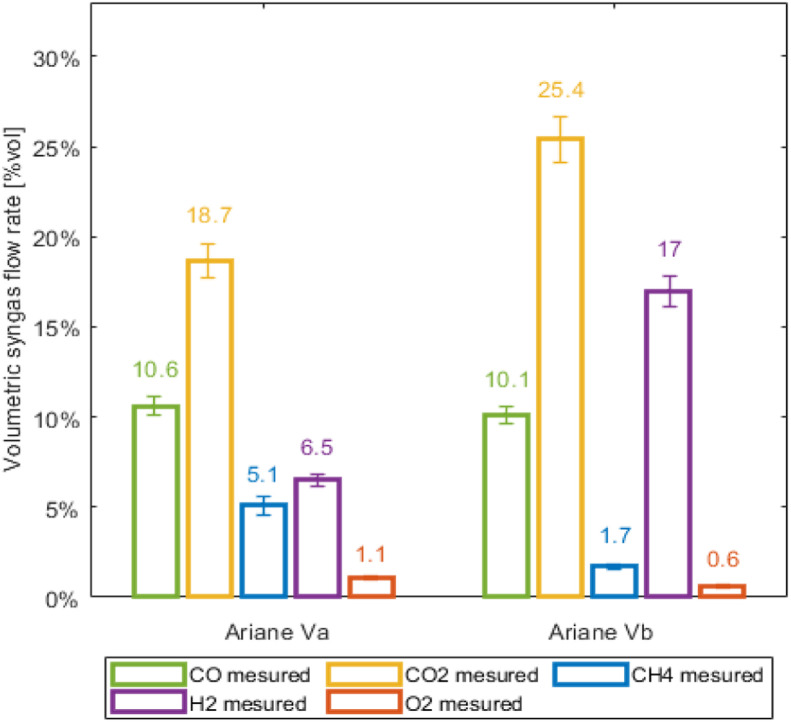
Syngas composition comparison depending on prototype version.

The reduction in the anhydrous yield of biochar is mainly due to the composition of the syngas, which has changed significantly compared with the previous version of the prototype (Fig. 3). The carbon-oxidising agent reactions enable the carbon to be converted into gas via a partial reduction stage. In both versions, the composition of the syngas in CO, $CO_{2}$, $H_{2}$ and $CH_{4}$ are not dependent on biomass flow rate.

Fig. 3 shows significant changes, mainly in the volume fractions of $CO_{2}$ and $H_{2}$ in the syngas. The volumetric $CO_{2}$ fraction increases by almost 7 points and that of $H_{2}$ by more than 10 points. However, these results must be set against a syngas flow rate that is approximately 10 % increased in the new version (since the air factor is slightly higher on the previous version). The $CH_{4}$ volumetric fraction also seems to decrease significantly in Vb.

### 3.3 Specific surface and carbon content

**Fig. 4:**
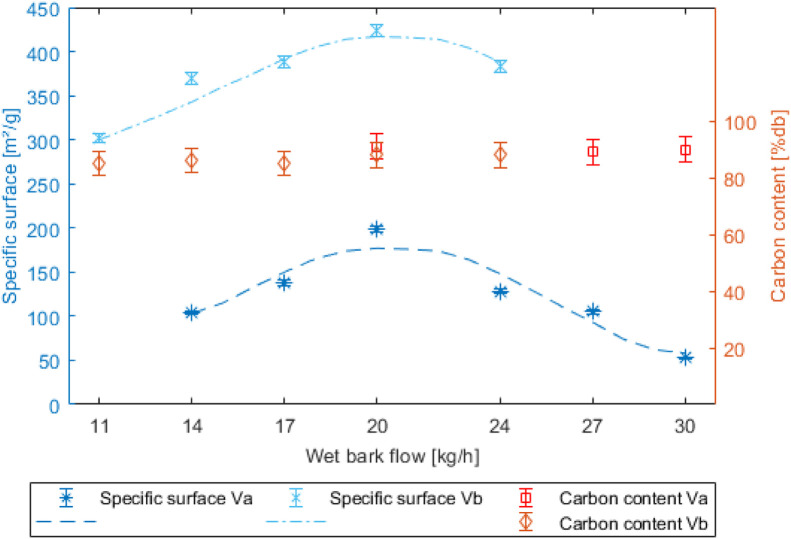
Specific surface and carbon rate depending on wet bark flow rate and prototype version.

Specific surface area and fixed carbon content are the main indicators of the potential agronomic quality of biochar. These two indicators are represented in Fig. 4. Specific surface area is measured by the BET method, which is the most used in the literature. The samples were analyzed consecutively to guarantee the reproducibility of the method.

The results obtained confirm the various observations made on the carbon conversion rate and biochar yield. These data are completely independent of the biomass flow rate. The specific surface measurement follows this trend, with an almost constant difference between the old and new versions of the pyrolyser, at around +250 $m^{2}/g$ thanks to the reduction zone allowing direct contact between biochar and syngas. The fluctuation in specific surface area as a function of biomass flow rate is therefore mainly due to pyrolysis conditions, since the two specific surface area curves follow each other in parallel. The optimum specific surface of 424 $m^{2}/g$ is obtained for the same raw bark flow rate, i.e. 20 kg/h.

The increase in specific surface area slightly reduces the fixed carbon content, but only to a small extent. The fixed carbon content of biochar fell by less than 5 points between the two versions of the pyrolyser. The fixed carbon rate is constant as a function of the biomass flow rate. As the fixed carbon rates between the versions and the tests were very close, the lower heating values (LHV) of biochars from the two versions are also contained between 30 and 32 MJ/kg.

## 4 Conclusion

The experimental phase of this work has made it possible to operate more fully the discoveries of Langlois [8] on the influence of the combination of syngas and hot biochar. This work indeed tested a method of in-situ activation by partial reduction of the post-pyrolysis biochar (which in general is done ex-situ, after cooling the biochar).

The experimental results are encouraging in terms of the operation and stability of the new version of the pyrolysis prototype with the addition of a reduction zone, as well as the quality of the biochar. The specific surface area grew from 200 to over 450$\;m^{2}/g$, while the carbon content remained stable at around 90 %, as did the ash content, which remained below 10 %. Although the reduction conditions are induced by the pyrolysis conditions and the partial oxidation of the gases in the furnace, the reduction temperature and the proportion of solid and gaseous components give the biochar improved properties compared with the old version of the pyrolyser without partial reduction of the biochar. The consumption of carbon from biochar (about 20 %) benefits the composition of syngas, with an increase in the volume fractions of $CO_{2}$ (from 19 to 25 %) and $H_{2}$ (from 7 to 17 %). A decrease in the level of $CH_{4}$ (from 5 to 2 %) is also observed. In the future, the aim is to open the prospects for this pyrolyser by testing other biomasses.

## CRediT authorship contribution statement

**Valentin Chataigner:** Writing – original draft, Methodology, Formal analysis, Data curation. **Dominique Tarlet:** Supervision, Software. **François Ricoul:** Supervision, Conceptualization. **Jérôme Bellettre:** Validation, Supervision, Funding acquisition.

## Declaration of competing interest

The authors declare that they have no known competing financial interests or personal relationships that could have appeared to influence the work reported in this paper.

## Data Availability

The data that has been used is confidential.

## References

[1] Kusuma H.S., Az-Zahra K.D., Saputri R.W., Utomo M.D.P., Jaya D.E.C., Amenaghawon A.N. (2024 Jun 1). Unlocking the potential of agricultural waste as biochar for sustainable biodiesel production: a comprehensive review. Bioresour. Technol. Rep..

[2] Urrutia R.I., Aagaard T.F., Gutierrez V.S., Werdin González J.O., Frechero M.A., Volpe M.A (2024 Jun 1). Co-production of bioinsecticide and biochar from sunflower edible oil waste: a preliminary feasibility study. Bioresour. Technol. Rep..

[3] Roy M., Kundu K. (2023 Jun 1). Production of biochar briquettes from torrefaction of pine needles and its quality analysis. Bioresour. Technol. Rep..

[4] Madrigal G., Huaraya M., Sancho T., Mendieta O., Jaimes-Estévez J. (2022 Dec 1). Biochar from bovine manure as a sustainable additive to improve the anaerobic digestion of cheese whey. Bioresour. Technol. Rep..

[5] Jaimes-Estévez J., Martí-Herrero J., Poggio D., Zafra G., Gómez K., Escalante H. (2023 Sep 1). The role of biochar in the psychrophilic anaerobic digestion: effects on kinetics, acids metabolism, and microbial population. Bioresour. Technol. Rep..

[6] Kohli A. (2022). Effets de composts autoproduits et d'un biochar sur le transfert d’éléments trace dans des légumes de jardins familiaux modérément contaminés [Internet] [phdthesis]. Agrocampus. Ouest..

[7] Chataigner V., Tarlet D., Ricoul F., Bellettre J. (2023). Experimental and theoretical study of heat and mass transfer in a continuous, vertical and coaxial pyrolysis reactor for high porosity biochar production. Fuel..

[8] Langlois S. (2019).

[9] Mermoud F., Golfier F., Salvador S., Van de Steene L., Dirion J.L. (2006). Experimental and numerical study of steam gasification of a single charcoal particle. Combust. Flame..

[10] Walker P.L., Rusinko F., Austin L.G., Eley D.D., Selwood P.W., Weisz P.B. (1959). Advances in Catalysis [Internet].

[11] Van de steene L., Tagutchou J.P., Escudero Sanz F., Salvador S. (2011). Gasification of woodchip particles: experimental and numerical study of char–H2O, char–CO2, and char–O2 reactions. Chem. Eng. Sci..

[12] Guizani C., Escudero Sanz F.J., Salvador S. (2013). The gasification reactivity of high-heating-rate chars in single and mixed atmospheres of H2O and CO2. Fuel..

[13] Wang Y., Kinoshita C.M. (1993). Kinetic model of biomass gasification. Sol. Energy..

[14] Chen G., Andries J., Luo Z., Spliethoff H. (2003). Biomass pyrolysis/gasification for product gas production: the overall investigation of parametric effects. Energy. Convers. Manag..

[15] Hernández J.J., Aranda-Almansa G., Bula A. (2010). Gasification of biomass wastes in an entrained flow gasifier: effect of the particle size and the residence time. Fuel. Process. Technol..

[16] Shayan E., Zare V., Mirzaee I. (2018). Hydrogen production from biomass gasification; a theoretical comparison of using different gasification agents. Energy. Convers. Manag..

[17] Jayaraman K., Gokalp I., Bonifaci E., Merlo N. (2015). Kinetics of steam and CO2 gasification of high ash coal–char produced under various heating rates. Fuel..

[18] Huo W., Zhou Z., Wang F., Wang Y., Yu G. (2014). Experimental study of pore diffusion effect on char gasification with CO2 and steam. Fuel..

[19] Puig-Arnavat M., Bruno J.C., Coronas A. (2012). Modified thermodynamic Equilibrium model for biomass gasification: a study of the influence of operating conditions. Energy. Fuels..

[20] Ghassemi H., Shahsavan-Markadeh R. (2014). Effects of various operational parameters on biomass gasification process; a modified equilibrium model. Energy. Convers. Manag..

